# Achieving Rapid Remission With Guselkumab in a Moderately Active Ulcerative Colitis Patient With High Infectious Risk and Inadequate Response to Advanced Therapy: A Case Report

**DOI:** 10.7759/cureus.107908

**Published:** 2026-04-28

**Authors:** Nozomi Furuichi, Yuki Itoi, Keiichi Oshima, Yoji Takeuchi, Toshio Uraoka

**Affiliations:** 1 Department of Gastroenterology and Hepatology, Gunma University Graduate School of Medicine, Maebashi, JPN

**Keywords:** advanced therapy inadequate response, guselkumab, il-23p19 antagonists, invasive candidiasis, ulcerative colitis, upadacitinib

## Abstract

Ulcerative colitis (UC) with prior inadequate response to advanced therapy (AT-IR) that requires hospitalization is particularly challenging to manage, especially in cases where a high risk of infection limits the use of immunosuppressive agents.

A 32-year-old male patient with pancolitis-type UC was refractory to glucocorticosteroids, infliximab, and tacrolimus. His clinical course was complicated by septic shock and invasive candidiasis. Antifungal therapy limited the use of upadacitinib due to drug-drug interactions. After achieving infection control, intravenous guselkumab (200 mg) was initiated. The patient’s stool frequency normalized, and hematochezia resolved within two weeks. The patient achieved clinical remission, and he was then discharged.

In conclusion, guselkumab achieved clinical remission in a patient with active UC with AT-IR, in whom the risk of infection was high and other advanced therapies were restricted.

## Introduction

Ulcerative colitis (UC) is a chronic inflammatory bowel disease (IBD) characterized by persistent inflammation of the colonic mucosa [1-3]. Oral or intravenous (IV) glucocorticosteroids (GCs) are generally used as the first-line therapy for moderately to severely active UC. In patients with resistance, intolerance, or contraindications to GCs, the use of biologics or small molecules should be considered. Treatment selection is individualized according to previous therapies, disease phenotype and severity, and patient characteristics. Even though advanced therapies are provided, UC with prior inadequate response to advanced therapy (AT-IR) is still challenging to manage [4-6].

Interleukin (IL) antagonists play an important role in current IBD management. Until recently, mirikizumab and risankizumab were the available IL-23p19 antagonists for UC. The efficacy of guselkumab was demonstrated in the QUASAR Study, and it was approved for UC management in 2024 [7]. However, the selection of guselkumab compared with other advanced therapies remains controversial.

Here, we report a case of a hospitalized male patient with moderately active UC with AT-IR, in whom recurrent infections limited therapeutic options. Thus, clinical remission was achieved with guselkumab. Although a single case, the clinical course might provide insights into the early postlaunch use of guselkumab.

## Case presentation

A 32-year-old male patient without a remarkable previous medical or family history presented with chronic abdominal pain and diarrhea. Seven months prior, the patient developed mild hematochezia, and colonoscopy performed at a local clinic confirmed pancolitis-type UC. Oral 5-aminosalicylic acid was initiated. However, it was discontinued due to intolerance caused by fever and increased bloody stools. Treatment with oral prednisolone (PSL) was initiated but failed to induce remission.

**Figure 1:**
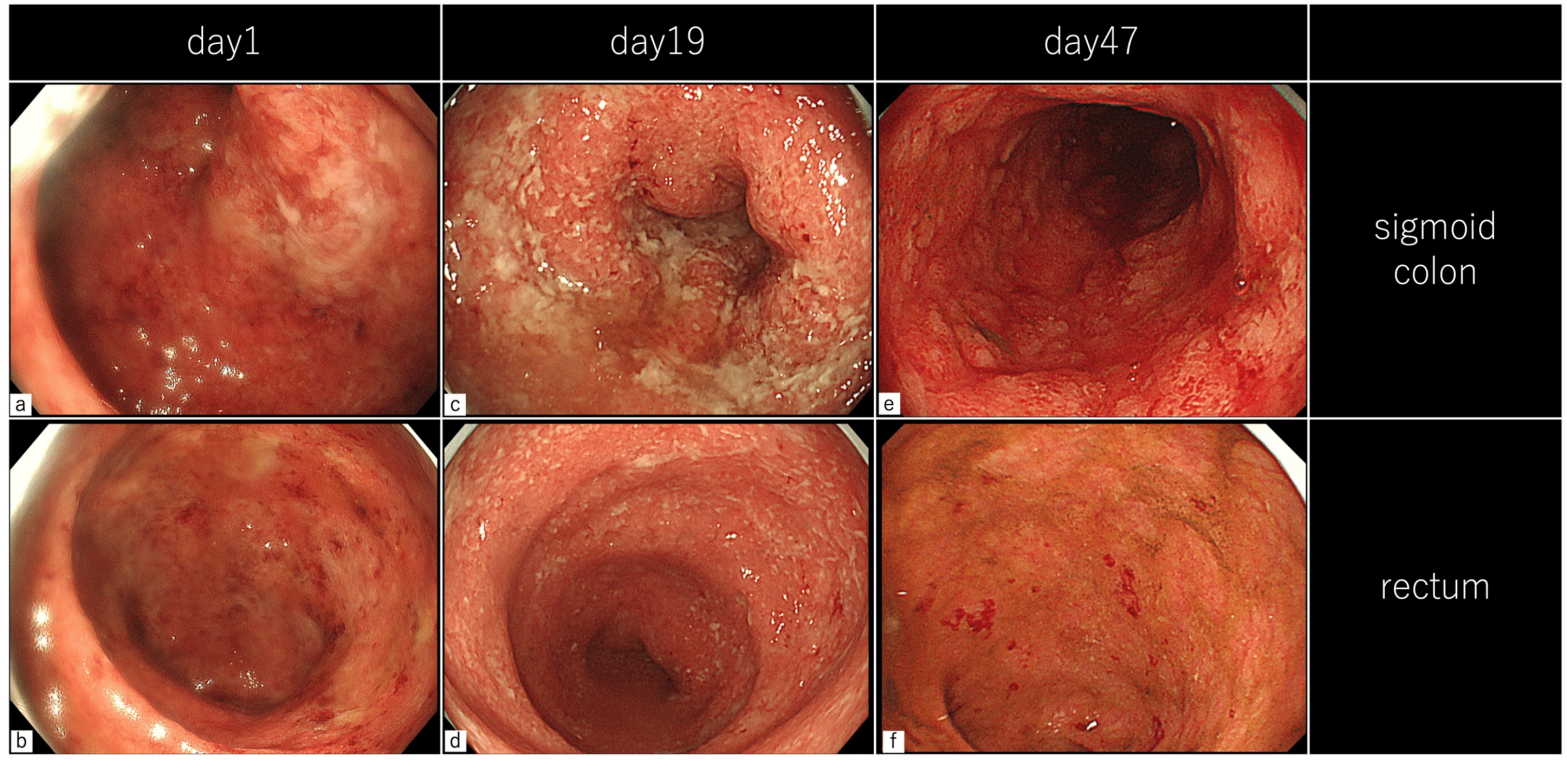
Colonoscopic findings at the previous hospital (a,b) After oral prednisolone 30 mg/day (c,d) After infliximab induction at 5 mg/kg (e,f) After tacrolimus at high trough levels Each examination showed a Mayo endoscopic subscore of 2, with persistent erythema, friability, and mucosal erosions despite improvement in purulent mucus and edema on day 47.

Hence, he was referred to the previous hospital. At that time, he presented with bloody stools 10 times daily. Colonoscopy showed a Mayo endoscopic subscore (MES) of 2 (Figure 1). He was admitted and received IV PSL (1 mg/kg). His condition did not improve. Thus, infliximab (IFX) was initiated at 5 mg/kg on day 10, and PSL was tapered. His primary response to IFX was poor, and tacrolimus (Tac) was started on day 38. During two weeks of high trough levels (10-15 ng/mL), the stool frequency temporarily improved to three times daily. However, hematochezia recurred at lower trough levels (5-10 ng/mL). All colonoscopies performed on days 1, 19, and 47 showed a persistent MES of 2. Due to ongoing anemia and intermittent fever, he was transferred to our department on day 67.

Upon admission, he had a fever with chills and rigors. His vital signs were as follows: temperature, 38.4°C; blood pressure, 77/57 mmHg; heart rate, 144 beats/min; and SpO₂ in room air, 98%. A central venous catheter (CVC) was inserted into the right internal jugular vein. Abdominal tenderness was minimal, and he passed soft, blood-tinged stools five times daily.

The laboratory examination findings ar shown in Table 1.

**Table 1:** The laboratory examination findings at the time of hospitalization Hematological analysis showed profound anemia. The total white blood cell count and platelet count were within the normal range. The inflammatory marker levels were not significantly elevated. The microbiological examination yielded negative results, including β-D-glucan. The lactate level was elevated.

Parameter	Value	Reference range
White blood cell count (WBC)	5.0 ×10³/µL	3.3-8.6
Neutrophils	38.5%	42.0-75.6
Lymphocytes	41.3%	17.4-48.2
Hemoglobin	6.0 g/dL	13.7-16.8
Platelet count	248 ×10^3^/µL	158-348
Total protein	7.1 g/dL	6.6-8.1
Albumin	2.7 g/dL	4.1-5.1
Total bilirubin	0.3 mg/dL	0.4-1.5
Aspartate aminotransferase (AST)	11 U/L	13-30
Alanine aminotransferase (ALT)	8 U/L	10-42
Lactate dehydrogenase (LDH)	105 U/L	124-222
Alkaline phosphatase (ALP)	72 U/L	38-113
Gamma-glutamyl transpeptidase (GGT)	20 U/L	13-64
Blood urea nitrogen (BUN)	4 mg/dL	8-20
Creatinine	0.69 mg/dL	0.65-1.07
Sodium	136 mEq/L	138-145
Potassium	4.3 mEq/L	3.6-4.8
Chloride	104 mEq/L	101-108
Ferritin	5.7 μg/L	14.4-303.7
Iron	10 μmol/L	40-188
Unsaturated iron binding capacity (UIBC)	268 μg/L	120-250
C-reactive protein (CRP)	7.1 mg/L	0.00-0.14
Procalcitonin	0.06 ng/mL	0.00-0.49
β-D-glucan	<3.3 pg/mL	0-20
Lactate	2.0 mmol/L	0.5-1.5
Erythrocyte sedimentation rate (ESR)	52 mm/h	2.0-10.0

**Figure 2:**
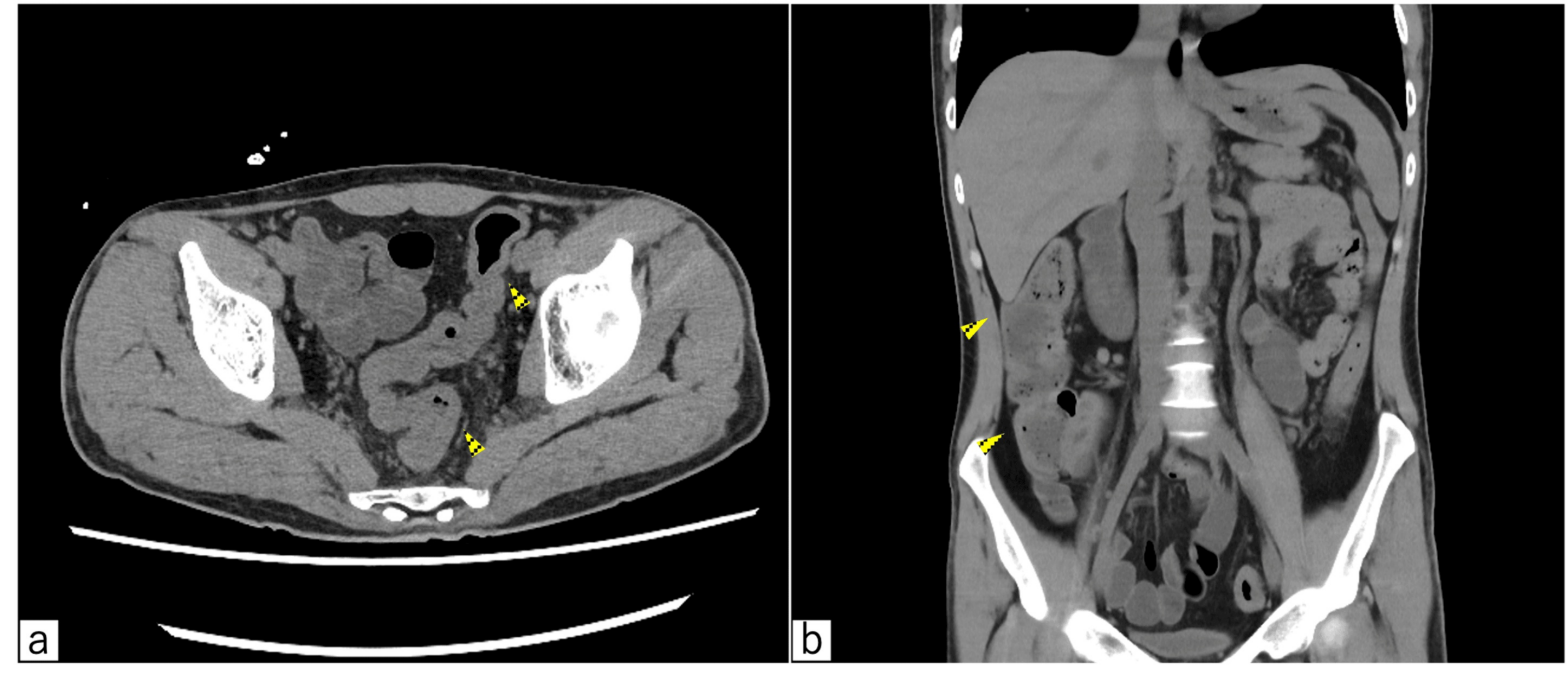
The plane CT scan at the time of hospitalization (a) The axial view of the pelvis The marker indicates thickening of the rectal wall. (b) The colonal view The marker indicates thickening of the right colonic wall. Diffuse thickening of the colonic wall extending from the rectum to the cecum, consistent with pancolitis-type UC. UC: Ulcerative colitis

Findings indicated severe iron deficiency anemia, hypoalbuminemia, no significant inflammation, and lactic acidemia. A computed tomography (CT) scan revealed diffuse colonic wall thickening without additional abnormalities (Figure 2). 

The patient developed a high fever and tachycardia. However, the endoscopic findings and abdominal symptoms were consistent with moderately active UC. Hypotension requiring vasopressors and an elevated lactate level fulfilled the diagnostic criteria for septic shock. A catheter-related bloodstream infection was suspected, and the existing CVC was removed.

The patient was admitted to the intensive care unit (ICU). Gram-positive cocci were observed on the blood smear, and empirical antimicrobial therapy with imipenem 1 g IV three times daily and vancomycin 1,500 mg IV once daily (then dose-adjusted) were initiated. Tac was discontinued to prioritize infection control. Packed red blood cells were transfused to maintain the hemoglobin level at ≥ 80 g/L. Then, his hemodynamics stabilized with the abovementioned treatments. He was discharged from the ICU on day 6. After blood cultures identified *Staphylococcus epidermidis*, imipenem was discontinued.

**Figure 3:**
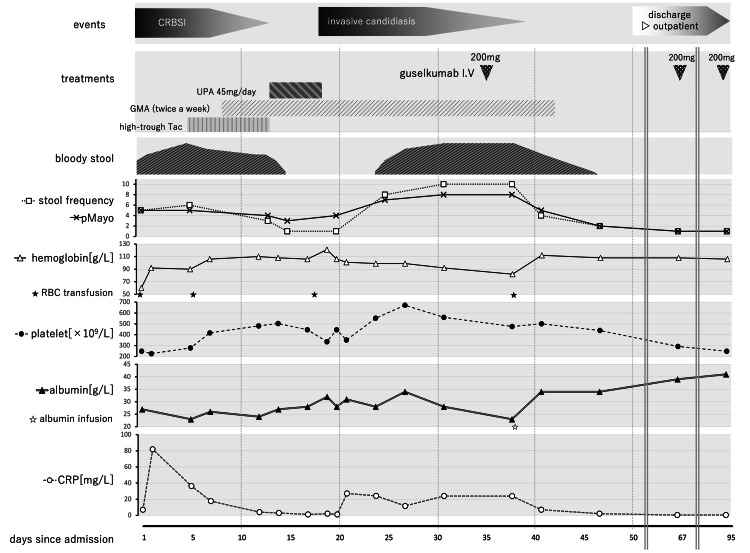
The clinical course after hospitalization The above is the timeline of infection events, granulocyte/monocyte adsorptive apheresis (GMA) and advanced therapies. Tacrolimus (Tac) was restarted on day 6, and switched to upadacitinib (UPA) on day 14 due to insufficient response. UPA was discontinued on day 18 owing to invasive candidiasis. In combination with antifungal treatment, guselkumab was initiated on day 35, leading to a rapid clinical improvement and discharge on day 50. CRBSI: Catheter-related bloodstream infection

At this point, hematochezia increased, and UC management was resumed. He was found to be refractory to GC. Moreover, he had primary nonresponse to IFX and partial response to Tac. Figure 3 shows the clinical course. Tac 5 mg/day was restarted on day 6 as a bridge therapy until infection control was secured. Starting on day 8, granulocyte/monocyte adsorptive apheresis (GMA) was initiated twice weekly.

As infection improved (as evidenced by β-D-glucan negativity, declining CRP levels, and two consecutive negative blood culture results), oral upadacitinib (UPA) 45 mg once daily was started, and Tac was discontinued on day 14. On day 16, hematochezia disappeared, and his stool frequency decreased to once daily, indicating a favorable response to UPA.

However, he developed a fever with recurrent rigors, and his β-D-glucan levels increased (21.3 ng/L) on day 18. Candidiasis was suspected, and micafungin 100 mg IV once daily was started empirically for severe candidiasis, and UPA was discontinued. On day 21, culture revealed micafungin-resistant Candida parapsilosis. Hence, the antifungal therapy was changed to fosfluconazole (F-FLCZ) IV (loading dose: 800 mg, followed by 400 mg daily). Ophthalmologic evaluation ruled out endophthalmitis. F-FLCZ was continued for two weeks from the negative blood culture results [8]. After F-FLCZ was initiated, his β-D-glucan levels declined rapidly. During this period, eight sessions of GMA was completed for UC therapy. However, both hematochezia and stool frequency worsened after UPA interruption. Restarting UPA was considered inappropriate due to the pharmacokinetic interaction with F-FLCZ, a potent CYP3A4 inhibitor [9]. Therefore, a reduction in the UPA dose was required.

After confirming the negative blood culture results on day 29, guselkumab 200 mg IV was administered on day 35. After a few days, hematochezia diminished, and the stool frequency decreased to four times a day. Diet was initiated on day 41, and GMA was completed on day 42 (with a total of 10 sessions). On day 46, the stool frequency normalized to two times a day, and the laboratory test results on day 48, which indicated improvement, were as follows: albumin level, 34 g/L and CRP level, 2.1 mg/L. Clinical remission was achieved within two weeks after guselkumab induction, and he was discharged to home on day 50.

**Figure 4:**
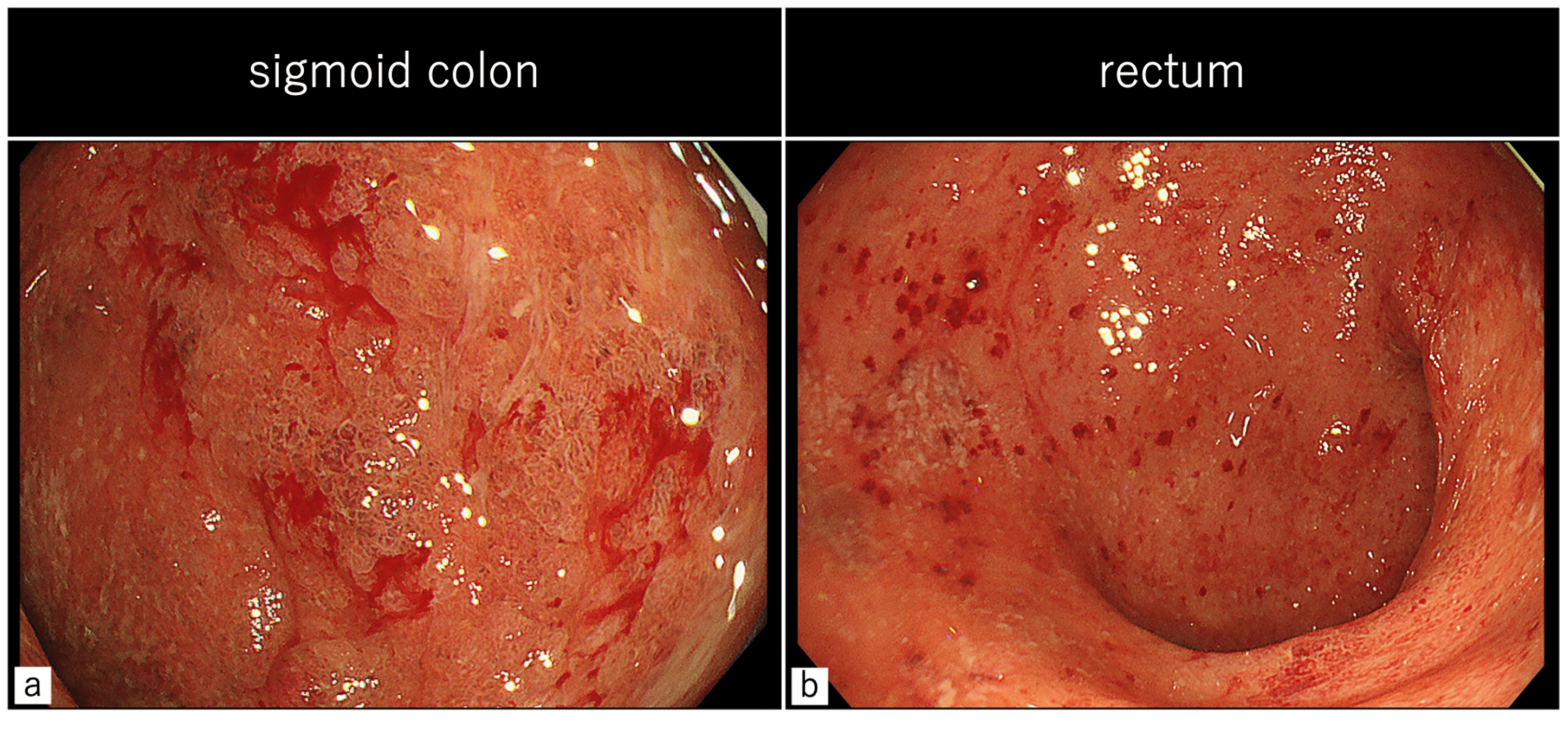
A colonoscopy at week 8 from induction of guselkumab Persistent erythema and mucosal erosions without ulcer healing were observed in the sigmoid colon and rectum (MES 2), indicating that endoscopic improvement had not yet been achieved.

After discharge, guselkumab 200 mg IV was administered again at week 4 and 8. He remained clinical remission. However, colonoscopy conducted at week 8 showed a MES of 2, indicating that endoscopic improvement requires additional time (Figure 4). Maintenance therapy with subcutaneous guselkumab 200 mg every four weeks is ongoing.

## Discussion

Guselkumab, an IL-23p19 antagonist, was selected due to several reasons. The patient in the current case had not previously received IL-12/23p40 or IL-23p19 antagonists, which have a favorable safety profile. Based on several studies, IL-23p19 antagonists have not been associated with an increased risk of infection, major adverse cardiovascular events, or malignancy compared with placebo [10]. In psoriasis, guselkumab is associated with a lower risk of major opportunistic infections compared with adalimumab [11].

Furthermore, guselkumab had a favorable efficacy in patients with moderately to severely active UC with AT-IR. In the phase 3 QUASAR study, guselkumab and placebo significantly differed across all evaluated endpoints, including clinical response, clinical remission, endoscopic improvement, and endoscopic remission, in both advanced therapy-naïve and AT-IR populations [7].

Due to differences in patient populations and endpoint definitions, direct cross-trial comparisons are not appropriate. Nevertheless, in the LUCENT-1 study (induction of mirikizumab), a statistically significant difference at week 12 was not observed for clinical remission in the AT-IR subgroup [12]. In the INSPIRE study (induction of risankizumab), the efficacy was generally favorable. However, a statistically significant difference was not found for endoscopic remission at week 12 in the AT-IR population [13].

Although UPA is recommended for moderately to severely active UC with AT-IR, guselkumab may be one of the therapeutic options when UPA cannot be used due to a risk of infection, drug-drug interactions, and various other reasons [14].

However, this was a unique case in which the clinical course was complicated by infection. Moreover, since endoscopic improvement had not been achieved at the initiation of maintenance therapy, it is necessary to monitor the course and confirm the efficacy of guselkumab over time. It represents a single-case observation. Future real-world data and prospective studies are required to identify optimal management strategies.

## Conclusions

In this case of a patient with moderately active UC who was refractory to GC, IFX, and Tac, and discontinued UPA due to drug-drug interaction, the initiation of guselkumab IV after control of invasive candidiasis led to rapid clinical remission within two weeks and sustained remission subsequently.

Guselkumab may be considered one of the therapeutic options for patients with moderately to severely active UC with AT-IR, particularly when UPA cannot be used because of a high risk of infection, potential drug-drug interactions, or other clinical considerations.

## References

[1] Danese S, Fiocchi C (2011). Ulcerative colitis. N Engl J Med.

[2] Ordás I, Eckmann L, Talamini M, Baumgart DC, Sandborn WJ (2012). Ulcerative colitis. Lancet.

[3] Lichtenstein GR, Rutgeerts P (2010). Importance of mucosal healing in ulcerative colitis. Inflamm Bowel Dis.

[4] Ananthakrishnan AN, Murad MH, Scott FI (2024). Comparative efficacy of advanced therapies for management of moderate-to-severe ulcerative colitis: 2024 American Gastroenterological Association Evidence Synthesis. Gastroenterology.

[5] Turner D, Ricciuto A, Lewis A (2021). STRIDE-II: an update on the Selecting Therapeutic Targets in Inflammatory Bowel Disease (STRIDE) initiative of the International Organization for the Study of IBD (IOIBD): determining therapeutic goals for treat-to-target strategies in IBD. Gastroenterology.

[6] Raine T, Bonovas S, Burisch J (2022). ECCO guidelines on therapeutics in ulcerative colitis: medical treatment. J Crohns Colitis.

[7] Rubin DT, Allegretti JR, Panés J (2025). Guselkumab in patients with moderately to severely active ulcerative colitis (QUASAR): phase 3 double-blind, randomised, placebo-controlled induction and maintenance studies. Lancet.

[8] Rex JH, Bennett JE, Sugar AM (1994). A randomized trial comparing fluconazole with amphotericin B for the treatment of candidemia in patients without neutropenia. Candidemia Study Group and the National Institute. N Engl J Med.

[9] Mohamed MF, Jungerwirth S, Asatryan A, Jiang P, Othman AA (2017). Assessment of effect of CYP3A inhibition, CYP induction, OATP1B inhibition, and high-fat meal on pharmacokinetics of the JAK1 inhibitor upadacitinib. Br J Clin Pharmacol.

[10] Bourgonje AR, Ungaro RC, Mehandru S, Colombel JF (2025). Targeting the interleukin 23 pathway in inflammatory bowel disease. Gastroenterology.

[11] Blauvelt A, Papp KA, Griffiths CE (2017). Efficacy and safety of guselkumab, an anti-interleukin-23 monoclonal antibody, compared with adalimumab for the continuous treatment of patients with moderate to severe psoriasis: results from the phase III, double-blinded, placebo- and active comparator-controlled VOYAGE 1 trial. J Am Acad Dermatol.

[12] D'Haens G, Dubinsky M, Kobayashi T (2023). Mirikizumab as induction and maintenance therapy for ulcerative colitis. N Engl J Med.

[13] Louis E, Schreiber S, Panaccione R (2024). Risankizumab for ulcerative colitis: two randomized clinical trials. JAMA.

[14] Singh S, Loftus EV Jr, Limketkai BN, Haydek JP, Agrawal M, Scott FI, Ananthakrishnan AN (2024). AGA living clinical practice guideline on pharmacological management of moderate-to-severe ulcerative colitis. Gastroenterology.

